# A Rare Case of Neck Abscess Caused by Salmonella

**DOI:** 10.7759/cureus.111818

**Published:** 2026-06-30

**Authors:** Melissa Cummins, Levi Stevens

**Affiliations:** 1 Otolaryngology - Head and Neck Surgery, West Virginia School of Osteopathic Medicine, Lewisburg, USA; 2 Otolaryngology - Head and Neck Surgery, West Virginia University, Martinsburg, USA

**Keywords:** diabetes, extraintestinal salmonellosis, incision and drainage (i&d), neck abscess, preventative care

## Abstract

Uncontrolled diabetes is a significant risk factor for infectious processes, many of which can present atypically. *Salmonella *frequently results in gastrointestinal infection, but direct inoculation or hematogenous spread can result in extraintestinal disease, a rare subset of which may include neck abscess. It is believed that patients with poorly controlled diabetes are predisposed to salmonellosis due to reduced gastric acidity and delayed gastric emptying, which promote survival and stagnation of the bacteria. This case demonstrates not only an otolaryngologic sequelae of uncontrolled diabetes, but the impact of care coordination and social determinants of health on medical outcomes for patients in rural and other underserved communities. We present the case of a 63-year-old man who arrived at the Emergency Department for medication reconciliation and was subsequently diagnosed with lymphadenitis. Conservative, stepwise management included oral antibiotics, ultrasound-guided aspiration and culture with broad-spectrum intravenous antibiotics, and ultimately incision and drainage. It is imperative in the diagnosis and management of individuals with atypical infections to consider predisposing conditions. In patients with hyperglycemia and neck abscess, *Salmonella *should be considered as a potential etiology. Additionally, timely, multidisciplinary care should be employed in order to coordinate medical management of comorbidities and improve compliance with preventative regimens while optimizing wound healing. Salmonellosis is a rare etiology of neck abscess that should be considered in patients with hyperglycemia. For those who do not respond to empiric regimens, culture sensitivity should direct definitive management.

## Introduction

*Salmonella* is a Gram-negative, flagellated bacillus of broad speciation, with over 1,800 known serovars exhibiting variable antibiotic susceptibility [1,2]. Antimicrobial resistance is increasing for the species, rising by nearly 15% over the course of seven years [3].

Infection with *Salmonella* spp. most commonly causes self-limited, cytokine-mediated gastrointestinal symptoms [1]. Extraintestinal manifestations are infrequent, occurring in ~1% of cases, and result predominantly from hematogenous spread via gastrointestinal mucosal invasion, or, less commonly, from direct inoculation [4]. For patients with diabetes, poor gastric mobility and reduced gastric acidity promote survival and stagnation of the bacterium, resulting in prolonged mucosal contact and an increased risk of hematogenous spread [4,5]. Although neck abscess is a rare endpoint of salmonellosis, it is more likely among diabetic patients, who have greater susceptibility to atypical infections in general [4]. Conversely, the most frequently identified etiologies of neck abscess among immunocompetent patients are odontogenic, with 90% of cultures revealing polymicrobial involvement [6].

## Case presentation

We present the case of a 63-year-old man with a past medical history of coronary artery disease (CAD), type 2 diabetes mellitus (T2DM), hyperlipidemia (HLD), hypertension (HTN), and cardiac stenting. He was referred to otolaryngology for a left submandibular mass after an initial presentation to the emergency department requesting a refill of medications for control of his diabetes and cardiac comorbidities, which he had not been able to access for four months due to relocating. During that time, his blood glucose had ranged from 250 to 300 on home readings. On exam, tender, left-sided lymphadenopathy was appreciated and had been gradually enlarging for one week. Associated symptoms included dysphagia, neck tenderness, excessive thirst, frequent urination, fatigue, and subjective fever. He denied any preceding history of traumatic inoculation to the area, including animal bite or scratch, and reported no recent travel.

On exam, the patient was hypertensive (155/99), tachycardic (110), and afebrile (37.1 °C). He was not toxic-appearing, and a poorly demarcated, left-sided mass without erythema or warmth was appreciated in the submandibular region. Initial imaging showed a 2.7 x 3.2 x 3 cm cystic, septated cervical lymphadenitis at level IB, IIA, with associated inflammatory changes in the left pharyngeal, tonsillar, and base of tongue regions (Figure 1). Laboratory evaluation demonstrated thrombocytosis (670 x 10^3/uL) consistent with acute phase reaction and hyperglycemia (355 mg/dL), without leukocytosis. Venous blood gases revealed normal pH (7.35) and no evidence of diabetic ketoacidosis. Urinalysis was remarkable for leukocyte esterase, nitrites, glucosuria >1000 mg/dL, ketonuria, and moderate bacteriuria suggestive of acute urinary tract infection.

**Figure 1:**
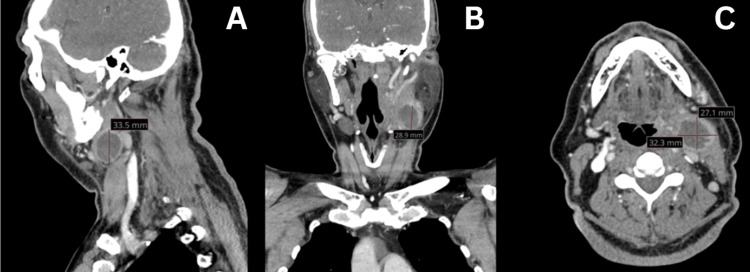
Initial contrasted CT imaging of 2.7 x 3.2 x 3 cm cystic, septated cervical lymphadenitis at level IBIIA in sagittal (A), coronal (B), and axial (C) planes. (A) Sagittal plane view of cystic lesion on CT scan; (B) coronal plane view of cystic lesion on CT scan; and (C) axial plane view of cystic lesion on CT scan.

At the time of discharge, the patient was placed on aspirin, glipizide, and empiric amoxicillin for management of his urinary tract infection and lymphadenitis, with subjective improvement initially. Upon presentation to the otolaryngology office four days after initial presentation, flexible nasopharyngoscopy revealed persistent asymmetry of the left arytenoid, aryepiglottic fold, piriform sinus, and tonsil.

He remained afebrile and normotensive, with asymmetrical tonsillar enlargement (3+ left, 1+ right), and persistent lymphadenopathy in the left level IBIIA nodes. Due to nonresponse to empirically prescribed amoxicillin, afebrile status, and absence of leukocytosis, the mass was not presumed to be of infectious etiology, and ultrasound-guided drainage was ordered to evaluate for possible branchial cleft cyst or malignancy, the latter of which was suspected. The patient experienced worsening dysphagia for the four days elapsing before the aspiration despite continued oral amoxicillin use. At the time of drainage, eight days after initial presentation, 20 mL of purulent fluid was collected, and the patient was admitted for intravenous vancomycin pending cultures of the aspirate (Figure 2).

**Figure 2:**
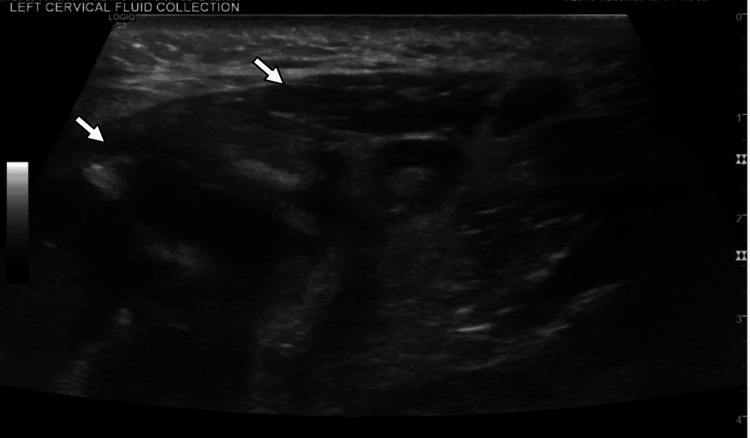
Ultrasound revealing multiloculated fluid collection in the neck, identified during ultrasound guided needle aspiration (white arrows). Subsequent ultrasound demonstrated improvement in the size of the fluid collection.

Thirteen days following his initial presentation, the patient underwent incision and drainage due to continued worsening on exam despite aspiration completed five days previously and appropriate escalation to intravenous vancomycin. Preoperatively, repeat CT revealed significant interval growth from 2.7 x 3.2 x 3 cm to 7.6 x 6.67 x 5.7 cm. The large fluid collection incorporated level IB-III lymph nodes, extended deep to the sternocleidomastoid muscle (SCM), and enveloped the submandibular gland with compression of the left supraglottic larynx and internal jugular vein (IJV), evidence of myositis in the SCM, and cellulitis of platysmal subcutaneous tissue (Figure 3).

**Figure 3:**
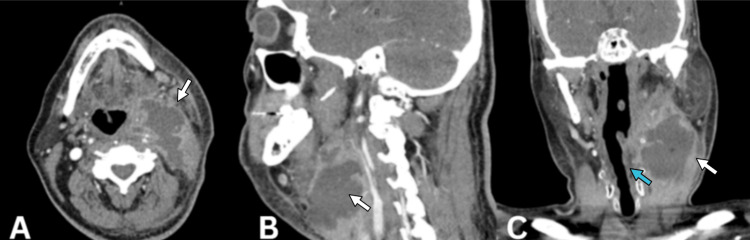
Contrasted CT imaging redemonstrating fluid collection in the axial (A), sagittal (B), and coronal (C) planes of the neck (white arrows), consistent with interval enlargement of the abscess and involvement of the platysma and SCM with compression and displacement of the larynx (blue arrow). (A) Axial plane view of cystic lesion on CT scan; (B) sagittal plane view of cystic lesion on CT scan; and (C) coronal plane view of cystic lesion on CT scan. SCM, sternocleidomastoid muscle.

During the procedure, 75 cc of purulent fluid was evacuated and cultured, and a 5 cm apron incision was left partially open and packed. Culture of the initial aspirate returned shortly after the procedure and grew *Salmonella* spp. with sensitivity to ampicillin (<=2), ceftazidime (<=0.5), ceftriaxone (<=0.25), ciprofloxacin (<=0.06), and levofloxacin (<=0.12). The patient was then discharged on a seven-day course of oral ciprofloxacin 500 mg in accordance with sensitivities in culture. Operative cultures would later redemonstrate the growth of pan-sensitive *Salmonella*.

Following the procedure, repeated flexible nasopharyngoscopy demonstrated interval improvement in edema. Wet-to-dry wound dressing was encouraged to debride the area. On continued follow-up, a 1 cm x 1 cm defect persisted at the incision site with mild superficial skin retraction, no purulence, and granulation tissue in place, which later fully approximated.

In the three months that elapsed from the time of initial presentation, the patient restarted insulin, had a diabetic foot and eye exam, was referred for and underwent colonoscopy, received shingles and pneumococcal vaccines, restarted lipitor, plavix, aspirin, and metoprolol, added an SGLT-2 inhibitor, ACE-Inhibitor, and GLP-1 to his regimen, and was referred to endocrinology and cardiology for maintenance of his medical comorbidities, in addition to becoming established with a primary care provider.

## Discussion

Salmonella is rarely a causative agent of neck mass, but should be considered among the differential diagnoses of hyperglycemic patients, particularly those who fail to respond to initial management. Atypical infections represent a potentially serious otolaryngologic sequela of poorly controlled diabetes. In this case, appropriate empiric selection and escalation of antibiotics with coverage of typical causative agents of deep neck infections, most commonly streptococcal and staphylococcal species, did not effectively target the causative agent [6]. Without consideration of atypical infectious processes among differential diagnoses in relevant patient demographics, delays in appropriate antibiotic selection pending cultures may prolong treatment durations and worsen clinical outcomes. In this case, progression of airway symptoms and compression of deep neck structures necessitated two interventional procedures, extended the duration of inpatient treatment, and increased healthcare-associated costs, while an appropriate antibiotic therapy was identified based on culture. A heightened degree of suspicion is particularly salient in areas where patients with poorly controlled diabetes are encountered commonly. For example, the incidence of T2DM and rates of poor glycemic control are significantly elevated among patients residing in areas with higher deprivation indices or rural composition [7]. Social determinants of health, including insurance status, income, proximity to healthcare providers, and access to transportation, should be recognized and addressed where possible, and multidisciplinary care should be coordinated in order to prevent complications of systemic disease and optimize wound healing [8]. Potential benefits for patients receiving multidisciplinary care include improved glycemic control and more frequent monitoring [8]. By improving glycemic control and preventing sequelae such as atypical infections, multidisciplinary management has the potential to reduce the need for emergent operative interventions, preserving healthcare resources and reducing associated costs and morbidity. This case, which highlights a sequelae of uncontrolled diabetes due to relocation, lack of transportation, and discontinuity of care due to scarce healthcare resources in a rural community, underscores the importance of mitigating these factors as well as the potential benefit of multidisciplinary care teams in promoting wellness.

## Conclusions

Frequent causative agents of neck infections include skin and oral flora, with odontogenic extension being the most prevalent etiology. However, atypical infection is possible, particularly among patients with immunocompromising comorbidities such as poorly controlled diabetes. Extraintestinal salmonellosis is a particularly rare etiology of neck abscess. Diabetes is a significant risk factor for salmonellosis as well as other atypical infections. In patients presenting with concurrent hyperglycemia or who fail to respond to empiric antibiotic therapy, the differential diagnosis should consider atypical microorganisms such as Salmonella. Suspicion should be further heightened among patients with a history of preceding gastrointestinal illness. For patients in whom salmonellosis is confirmed via culture, clinicians should maintain a high degree of suspicion regarding resistance, and culture-derived sensitivities should direct treatment selection. Antibiotic resistance is rising for the species, highlighting the importance of antimicrobial stewardship and targeted therapy using the narrowest possible antibiotic agent. Furthermore, all patients should be counseled on the importance of completing the full course of their prescription. Additional patient education should emphasize the importance of continued diabetes management in preventing complications such as atypical infections. Further preventative benefit can be realized through the use of multidisciplinary care teams to achieve superior glycemic control, shorter interval follow-up, and improved care continuity, particularly for patients in rural or underserved areas.
